# Apelin Protects Primary Rat Retinal Pericytes from Chemical Hypoxia-Induced Apoptosis

**DOI:** 10.1155/2015/186946

**Published:** 2015-09-27

**Authors:** Li Chen, Yong Tao, Jing Feng, Yan Rong Jiang

**Affiliations:** ^1^Department of Ophthalmology, People's Hospital, Peking University, 11 Xizhimen South Street, Xicheng District, Beijing 100044, China; ^2^Key Laboratory of Vision Loss and Restoration, Beijing Key Laboratory of Diagnosis and Therapy of Retinal and Choroid Diseases, Ministry of Education, 11 Xizhimen South Street, Xicheng District, Beijing 100044, China

## Abstract

Pericytes are a population of cells that participate in normal vessel architecture and regulate permeability. Apelin, as the endogenous ligand of G protein-coupled receptor APJ, participates in a number of physiological and pathological processes. To date, the effect of apelin on pericyte is not clear. Our study aimed to investigate the potential protection mechanisms of apelin, with regard to primary rat retinal pericytes under hypoxia. Immunofluorescence staining revealed that pericytes colocalized with APJ in the fibrovascular membranes dissected from proliferative diabetic retinopathy patients. In the *in vitro* studies, we first demonstrated that the expression of apelin/APJ was upregulated in pericytes under hypoxia, and apelin increased pericytes proliferation and migration. Moreover, knockdown of apelin in pericyte was achieved via lentivirus-mediated RNA interference. After the inhibition of apelin, pericytes proliferation was inhibited significantly in hypoxia culture condition. Furthermore, exogenous recombinant apelin effectively prevented hypoxia-induced apoptosis through downregulating active-caspase 3 expression and increasing the ratio of B cell lymphoma-2 (Bcl-2)/Bcl-2 associated X protein (Bax) in pericytes. These results suggest that apelin suppressed hypoxia-induced pericytes injury, which indicated that apelin could be a potential therapeutic target for retinal angiogenic diseases.

## 1. Introduction

Pericytes are a population of contractile cells that surround the endothelial cells of microvessel [1]. Genetic studies have shown that pericytes exert multiple effects on the vasculature: they participate in vascular development, maturation, and remodeling, and they also contribute to normal architecture and regulate permeability [1–3]. Retina, a light sensitive layer lining at the back of the eye, has the highest pericyte density around the body [4]. Recently, pericytes have received considerable attention as an active player in the pathological mechanisms of retinal angiogenic diseases, such as diabetic retinopathy (DR) and retinopathy of prematurity (ROP) [5, 6]. DR is one of the most common and important chronic microvascular complications in individuals with diabetes mellitus, which will give rise to blindness in uncontrolled conditions. Numerous studies have shown that the primary morphological change in the diabetic retina is the dysfunction and loss of pericytes [7]. The absence of pericytes destabilizes retinal vessels, making them more susceptible to hypoxia-induced sprouting [7, 8]. Others showed that pericytes are also involved in pathological retinal angiogenesis in a murine model of ROP [9]. Therefore, pericytes are currently under consideration as therapeutic targets for the treatment of retinal angiogenic diseases.

Apelin, a natural ligand for an orphan G protein-coupled receptor APJ, is widely expressed in various tissues, including brain, heart, lung, kidney, uterus, and ovary [10, 11], and is reported to be involved in the regulation of multiple physiological functions [12]. The studies of apelin-deficient mice and Xenopus laevis embryos indicate that apelin is involved in the regulation of vasculogenesis and angiogenesis [13, 14]. Hara et al. showed that the size of choroidal neovascular membrane lesions was decreased in apelin gene knockout mice [15], and* in vitro* studies have shown that apelin induced proliferation and migration of vascular smooth muscle cells (VSMCs) and suppresses VSMCs apoptosis induced by serum deprivation via APJ/PI3-K/Akt signaling pathways [16, 17]. In endothelial cells apelin also significantly enhanced migration, proliferation, and capillary-like tube formation [18]. Besides, our previous studies showed that apelin significantly enhanced the viability, migration, and proliferation of Müller cells and retinal pigment epithelium (RPE) cells through the pathway of MAPK/Erk and PI3-K/Akt [19–21].

Hypoxia has been widely used as a typical apoptosis insult to a variety of cell types [22]. Hypoxia has been shown to induce rat pancreatic *β*-cell apoptosis through Bcl-2/Bax pathway [23]. Previous studies demonstrated that apelin suppressed apoptosis in osteoblastic cell, human osteoblasts, and bone marrow mesenchymal cells through regulation of Bcl-2/Bax via PI3-K pathway [24–26]. Moreover, apelin reduced cytochrome c release from mitochondria to cytoplasm and activation of caspase 3. These results explained apelin protected cells via various mechanisms.

However, whether apelin has protective effects on rat primary pericytes has not been explored. We propose a putative role for apelin in the viability and apoptosis of pericytes and conducted this study to investigate whether apelin could exert protective effects on primary rat retinal pericytes under hypoxia.

## 2. Materials and Methods

### 2.1. Reagents

Exogenous recombinant apelin-13 peptide was purchased from Sigma (St. Louis, MO). The makers of pericytes were PDGFR-B (ab69506, Abcam, US), NG2 (SC-20162, Santa Cruz, CA), and Desmin (ab6322, Abcam, US). Antiapelin and anti-APJ were purchased from Abcam company (ab59469, ab125213, and ab84296, Abcam, US), respectively. CellTiter 96 AQueous One Solution was used in cell Proliferation Assay (Promega, US). Lentiviral vector knockout of apelin was constructed and purchased from GeneChem Co., Ltd. (Shanghai, China). Bcl-2 and Bax antibody were obtained from Cell Signaling Technology (#2870; #2772, CST, US).

### 2.2. Primary Rat Pericyte Cells Isolation, Culture, and Treatment

All experiments were performed in accordance with the Research Ethics Committees of the People's Hospital, Peking University, China. We isolated primary rat retinal pericyte cells from the retinal microvessel of Sprague-Dawley (SD) rats, using a modified method of previously published articles [27–29]. Briefly, eyes from SD rats (4–6 weeks, 150–200 g) were incubated with cold Dulbecco's phosphate-buffered saline (PBS) containing penicillin-streptomycin antibiotic (500 U/mL) for 10 min. The retinas were removed and cut into 1 × 1 mm small pieces and then incubated with collagenase I (Roche Applied Science, Mannheim, Germany) for 30–45 min at 37°C. The digested retina were filtered through 70 *µ*m and 40 *µ*m nylon mesh (Falcon, BD, US) and then centrifuged. Rat retinal pericytes were purified with Dynabeads Pan Mouse IgG (Invitrogen Dynal AS, Norway) according to the instructions. Before use, we washed the Dynabeads (25 *µ*L) in Dulbecco's Modified Eagle's Media (DMEM) (Hyclone, US), and we added 1 *µ*L mouse anti-desmin monoclonal antibody (ab6322, abcam, US) and then incubated them overnight at 4°C. The cell pellets were suspended in DMEM containing 10% fetal bovine serum (Gibco, US) and incubated with Dynabeads conjugated mouse anti-desmin monoclonal antibody for 30 min at 37°C, with gentle rotation. After washing, the bead-bound pericytes in pericyte medium (Sciencell Inc., US) were suspended at 37°C in a humidified atmosphere of a 5% CO_2_ incubator. Pericytes between passages three and five were used throughout the study.

In chemical hypoxia-induced pericytes injury models, 150 *µ*mol CoCl_2_ was used according to the previous report [21]. In the viability assay, we treated pericytes with different concentrations of apelin (10, 100, and 1000 ng/mL) and knockdown of apelin was performed via lentivirus vector. We incubated control group cells in pericyte medium.

### 2.3. Immunofluorescent Staining

Immunofluorescent staining of membranes tissue was described in our previous published study [20, 30]. Briefly, 12 fibrovascular membranes with proliferative diabetic retinopathy were surgically obtained during vitrectomy. In a similar manner, 10 macular preretinal membranes were obtained and served as control. Immunofluorescence staining was performed on the frozen sections of the fibrovascular membranes and of the control membranes by staining with rabbit anti-apelin (ab59469, 1 : 100, Abcam, US) or rabbit anti-APJ (ab84296, 1 : 200, Abcam, US). The human patients study protocol was approved by the Ethical Committee and Institutional Review Board of Peking University People's Hospital (Beijing, China) and was conducted in accordance with the Declaration of Helsinki. Written informed consent was obtained from each study subject.

Pericytes which were cultured on cover slides (Fisher, US) were fixed with 4% paraformaldehyde and incubated in 0.3% H_2_O_2_ and 0.1% triton X-100 to quench endogenous peroxidase activity and penetrate the cytomembrane. Then, the cells were incubated in 3% blocking goat serum for 1 h and then incubated with anti-PDGFR-*β* (ab69506, 1 : 100, Abcam, US), NG2 (SC-20162, 1 : 100, Santa Cruz, CA), desmin (ab6322, 1 : 200, Abcam, US), apelin (1 : 100, Abcam, US), and APJ (1 : 200, Abcam, US) overnight at 4°C. The following day, pericytes were incubated with the relevant fluorescence-conjugated secondary antibody (1 : 200, Invitrogen, US) for 2 h at room temperature. Images were obtained with Nikon 50i fluorescent microscope (Nikon, Tokyo, Japan) under 200x magnifications.

### 2.4. Lentivirus-Mediated shRNA Knockdown of Apelin Expression and Transfection

The knockdown of Apelin (Rattus, NM_031612.2, GI:52345441) was induced by a lentivirus-mediated RNA interference vector (GeneChem Co., Ltd., Shanghai, China). The small interfering RNA (siRNA) target sequences were selected: #1, 5′-GAGGAGAGATAGAAACAGA-3′; #2, 5′-GGAGGATGTTGGCTGAGAA-3′; #3, 5′-GTTTGCCTTTCTTGACAAA-3′; and #4, 5′-CAGATGAGTTCTCTTCTCT-3′. The lentivirus-GFP (LV-GFP) which included the GFP gene and did not include the apelin interference sequence served as negative control. For lentivirus transduction, pericytes were cultured at 5 × 10^4^ cells/well into 6-well culture plates. After being grown to 70% confluence, cells were transduced with shRNA lentivirus at a multiplicity of infection (MOI) of 100. Cells were harvested at 24 hours after infection, and transfection efficiency was evaluated by immunofluorescence staining. The knockdown efficiency of apelin was evaluated by RT-PCR and western blot analysis.

### 2.5. Cell Viability/Cell Proliferation

Pericyte viability was measured by MTS assay, according to the manufacturer's instructions (CellTiter 96 AQueous One Solution Assay; Promega, Madison, WI, US). 5000 cells/well were seeded into a 96-well plate and incubated with different concentrations of apelin (1, 100, and 1000 ng/mL) for 24 h. At the end of the incubation, 10 *µ*L MTS solution was added into each well and incubated for 1 h. The absorbance wavelength was evaluated with microculture plate reader (Model 550; Bio-Rad, Tokyo, Japan) at 490 nm (OD_490_). Each experiment was performed in five wells and repeated at least three times.

### 2.6. Edu Assay

Pericytes proliferation was assessed using a Cell-Light EdU Apollo 643* in vitro *Imaging Kit (RuiBo. Inc., Guangzhou, China). Briefly, 1 × 10^4^ cells/well, which was pretreated with apelin, was plated in 96-well plates for 24 h. Following the incubation interval, 10 *µ*mol 5-ethynyl-2′-deoxyuridine (Edu) medium was added to each well and incubated for 2 h. After washing twice with PBS, pericytes were fixed with 4% paraformaldehyde for 30 min and washed with 2 mg/mL glycine solution for 5 min in order to neutralize paraformaldehyde and assure a good staining system. The cells were incubated in 100 *µ*L 1x Apollo staining solution for 30 min and the nuclei were dyed with Hoechst 33342. The images were obtained under 10x magnification, using a Nikon 50i fluorescent microscope. Each experiment was performed in five wells and repeated at least three times.

### 2.7. Cell Migration/Transwell Assay

Transwell assay was used for evaluating cell migration assays. Briefly, 100 *µ*L of pericyte suspension (1 × 10^5^ cells/mL) was added to the upper chamber and 600 *µ*L medium containing apelin, hypoxia medium, or DMEM (control) to the lower chamber, respectively. The chambers were incubated for 6 h at 37°C. The filters were fixed with 4% paraformaldehyde for 15 min and we subjected the nuclei to DAPI staining for 10 min. The remaining cells on the upper surface of the filter were removed by wiping with a cotton swab. The number of migrated cells were quantified by counting in five random fields (10x magnification), using a Nikon 50i fluorescence microscope. The data are shown as the mean ± standard deviation (SD). Each experiment was repeated at least three times.

### 2.8. TUNEL Assay

Pericyte apoptosis after hypoxia was evaluated by terminal deoxynucleotidyl transferase-mediated dUTP nick end-labeling (TUNEL) assay. TUNEL staining was performed on cell coverslips using a commercial kit (In Situ Cell Death Detection Kit; Roche Applied Science, USA), according to the manufacturer's recommended instructions. TUNEL-positive cell nuclei were visualized as green fluorescence and images performed under 10x magnification. Finally, the percentage of TUNEL-positive cells was calculated in five microscopic fields of each slide.

### 2.9. Quantitative Real-Time PCR

Total RNA was isolated from cultured pericytes using Trizol reagent (Invitrogen, CA, US), and we determined the concentration and integrity of total RNA with UV spectrophotometry (NANODROP 2000C, Thermo, US). We used the Fermentas reverse transcription system (Fermentas, St. Leon-Rot, Germany) to reverse RNA (1 *μ*g) into first strand cDNA, using a real-time PCR system (PikoReal 96 PCR system, Thermo Scientific). The PCR solution system contained 1 *µ*L of cDNA (1 : 20 diluted), specific primers 1 *µ*L (10 pmol), 3 *µ*L DEPC-water, and 5 *µ*L of SYBR Select Master Mix (Invitrogen), with a final volume of 10 *µ*L. Each sample was measured in triplicate wells. Primers used were as follows: *β*-actin Forward: 5′-TGGCTCTATCCTGGCCTCACT-3′, *β*-actin Reverse: 5′-GCTCAGTAACAGTCCGCCTAGAA-3′; rat apelin Forward: 5′-GATGGAGAAAGGCGAAGAAAG-3′, rat apelin Reverse: 5′-GGTGAGAGATGAGACCACTTGT-3′. The standard PCR conditions included 2 min at 50°C and 10 min at 95°C, followed by 35 cycles of extension at 95°C for 15 s, 60°C for 30 s, and 72°C for 30 s. The mRNA expression was normalized to the expression level of ACTB. We calculated the changes in mRNA expression according to the 2^−ΔΔ^CT method, with ΔCT = C_Target  gene_ − CT_ACTB_ and ΔΔCT = ΔC_Treatment_ − ΔCT_Control_. Each experiment was repeated at least three times.

### 2.10. Western Blot Analysis

Pericytes were harvested and lysed in RIPA buffer (1% Nonidet P-40, 0.5% sodium deoxycholate, 0.1% SDS in PBS) and centrifuged at 15,000 rpm for 15 min at 4°C. The membranes were blocked with 5% nonfat milk for 1 h and then incubated overnight at 4°C with primary antibody: rabbit anti-Bcl-2, (#2870; 1 : 1000, CST); rabbit anti-Bax, (#2772; 1 : 1000; CST); rabbit anti-Apelin (ab125213; 1 : 500; abcam); and rabbit anti-APJ (ab84296; 1 : 1000, abcam). The membranes were incubated with goat anti-rabbit horse-radish peroxidase- (HRP-) conjugated secondary antibody (1 : 3000, DAKO, Japan) for 1 h at room temperature. The density of each band was analyzed with Image J software. Each experiment was repeated at least three times.

### 2.11. Statistical Analysis

The results were expressed as mean ± SD. Difference between two groups was compared with an independent sample* t*-test (SPSS17.0 software, Chicago, IL). Two-tailed *P* < 0.05 was considered to indicate statistical significance. Differences among groups were assessed using one-way analysis of variance (ANOVA), followed by Dunnett's test. A value of *P* < 0.05 was considered as significantly different. We repeated all experiments at least three times, and representative experiments are shown.

## 3. Results

### 3.1. Immunohistochemical Expression of APJ in Fibrovascular Membranes

**Figure 1:**
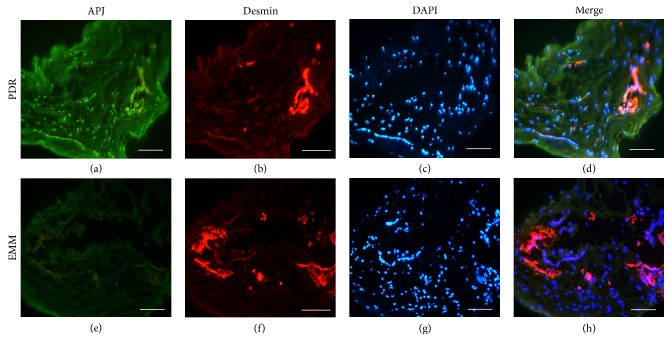
Immunostaining of APJ and pericyte in fibrovascular membranes. (a–d) Immunostaining for APJ (a), pericyte marker desmin (b), and DAPI (c) in fibrovascular membranes from eyes with proliferative diabetic retinopathy. Staining intensities of APJ were strong and were colocalized with pericyte, as identified by desmin. (e–h) Staining of APJ (e), desmin (f), and DAPI (g) in epiretinal macular membranes (EMM) of control patients without diabetic retinopathy. None of APJ (e) and staining of desmin (f) were observed. Scale bar = 100 *μ*m.

Expression of APJ was detected in the specimens of all fibrovascular membranes of the proliferative diabetic retinopathy (PDR) group with strong staining for APJ (Figure 1). Colocalization of pericyte markers desmin and APJ were observed in all specimens of the PDR group (*n* = 12) (Figures 1(a)–1(d)). None of the membranes removed from the eyes of the epithelial macular membrane (EMM) group showed specific staining of APJ (*n* = 10) (Figures 1(e)–1(h)). Our previous study also demonstrated that vitreous concentrations of apelin were significantly higher in the PDR group than in the EMM group [30].

### 3.2. Cultivation and Identification of Primary Rat Retinal Pericytes

**Figure 2:**
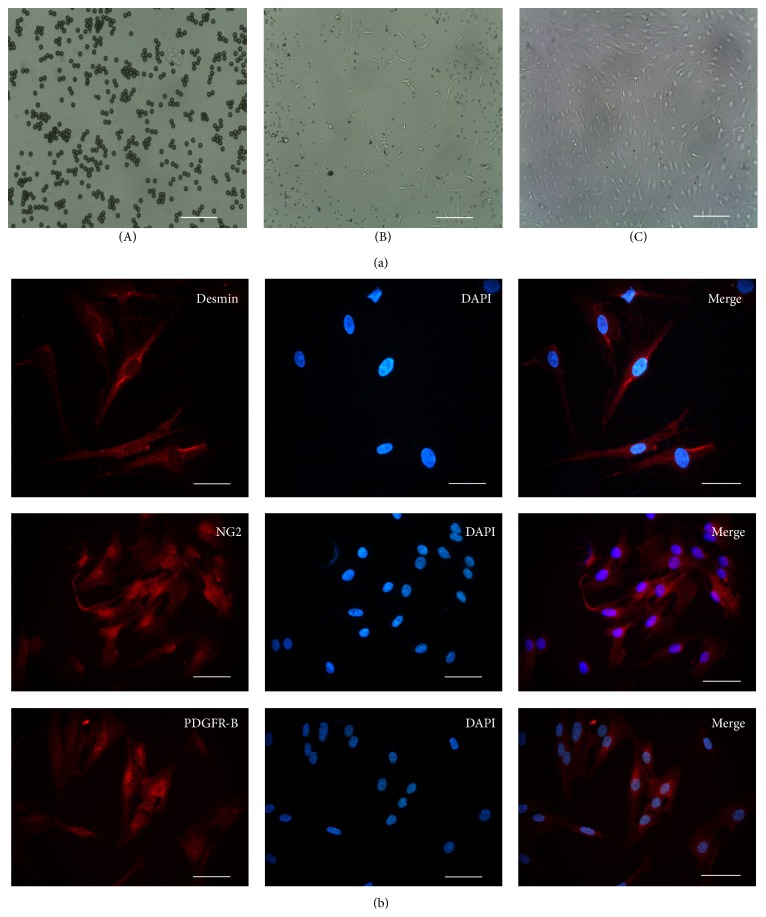
Morphology and immunofluorescent staining were identified in primary rat retinal pericytes. (a) Microscopic image of retinal pericytes, showing cell mass isolated by Dynabeads (A), at 7 days, pericytes got adherence and formed cells cluster (B), at passage 1 non-contact-inhibited growth of pericyte and irregular triangular cell bodies, with thick filaments in the cytoplasm and a plump nucleus (C). Scale bar = 200 *µ*m. (b) Immunofluorescence staining with anti-desmin, NG2, and PDGFR-B antibody for primary rat retinal pericytes, respectively. Scale bar = 100 *µ*m.

**Figure 3:**
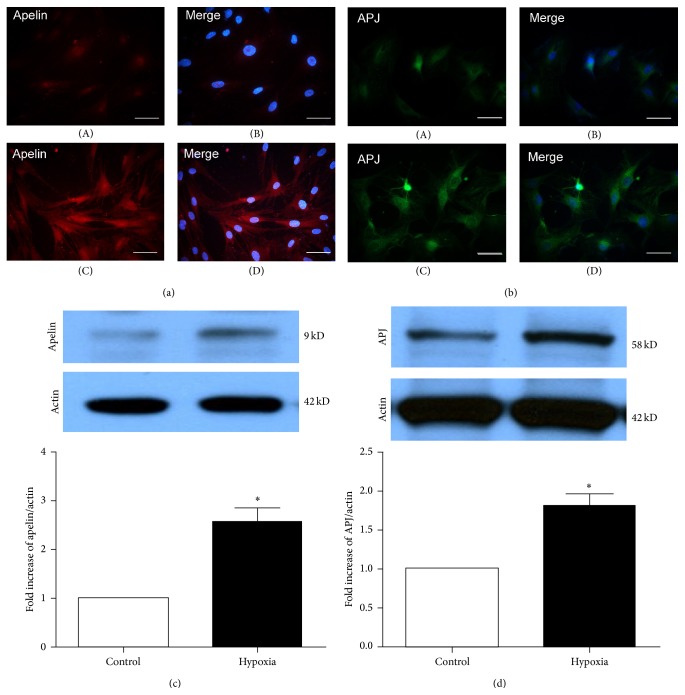
The cellular localization of apelin/APJ. (a) Apelin immunoreactivity was found weak and diffused in the cytoplasm of normal (A) but showed more intense cytoplasmic staining in hypoxic pericyte (C). Scale bar = 100 *μ*m. Similarly, (b) compared with restricted cytomembrane expression in normal (A) and hypoxia (C) pericyte, APJ localization expanded and brightened in the cytoplasm and cytoplasm membrane (apelin in red, APJ in green, and DAPI in blue). Scale bar = 100 *μ*m. (c) and (d) Western blot analysis shows that the expression of apelin and APJ under hypoxia was upregulated 2.5-fold and 1.9-fold, respectively (*P* < 0.05).

Primary rat retinal pericytes were isolated by Magnetic Dynabeads and formed cell clusters floating in cell medium (Figure 2(A)). At day 7, the primary pericytes got adherence, and the colony formed and grew (Figure 2(B)). Generally, primary pericytes confluenced at about day 14. When pericytes were passaged, the growth and adherence became rapid obviously, which got adherence about 4–6 hours and passaged about 4-5 days. Primary rat retinal pericytes showed irregular triangular cell bodies, with thick filaments in the cytoplasm and a plump nucleus (Figure 2(C)). As specific markers for pericyte, desmin, PDGFR-*β*, and NG2 were used to confirm the purity of cultured primary rat pericyte cells, which was approximately 95% (Figure 3(b)).

### 3.3. Expression of Apelin and APJ Receptor in Pericytes

Prior to exploring the effects of apelin in rat retinal pericytes, we carried out immunofluorescence staining to detect the expression of apelin/APJ in pericytes. We observed low apelin immunoreactivity in normal pericyte culture, which showed weak and diffuse expression in the cytoplasm (Figure 3(a)-(A), (B)). In similar way, the APJ staining was moderate, which was expressed in cytoplasm membrane (Figure 3(b)-(A), (B)). However, after exposure under hypoxia for 12 h, the expression of apelin represented obvious stronger cytoplasm staining (Figure 3(a)-(C), (D)), accompanied by expanding stronger APJ immunoreactivities in cytoplasm and cytoplasm membrane (Figure 3(b)-(C), (D)). In addition, the results of western blot about apelin and APJ under hypoxia support the change of immunofluorescence staining (Figures 3(c) and 3(d)). The expression of apelin and APJ under hypoxia was upregulated 2.5-fold and 1.9-fold, respectively (*P* < 0.05).

### 3.4. Detection of Interference Efficacy of Lentivirus-Apelin

**Figure 4:**
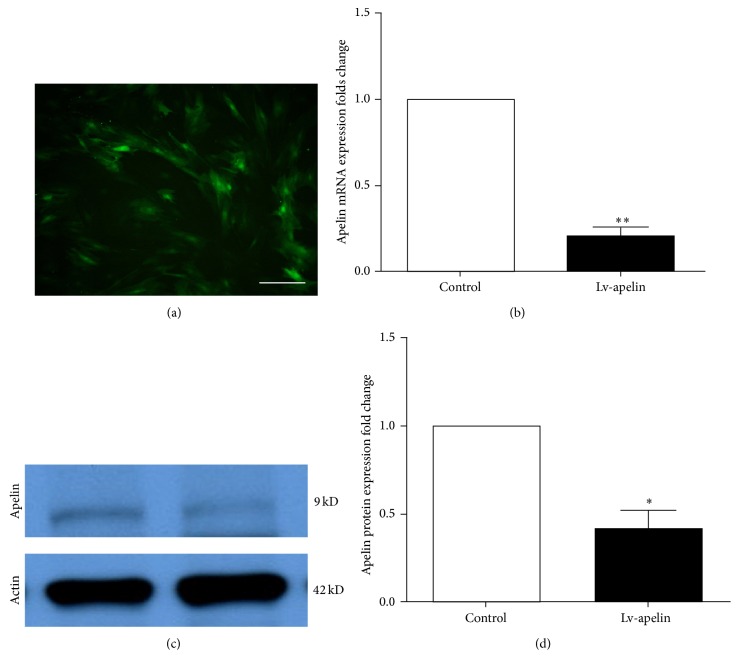
Pericytes transducted with Lentivirus-Apelin. (a) Immunofluorescence staining of LV-apelin infection in pericyte. Interference efficacy arrived about 90%, MOI = 100. Scale bar = 100 *µ*m. (b) The mRNA expression of LV-Apelin was decreased by 75% after blocking by siRNA sequence (*P* < 0.01). (c and d). The western blot analysis shows that protein of expression of LV-Apelin decreased by 64% (*P* < 0.05).

Quantitative Real-Time PCR in NRK-52E and IEC6 cells demonstrated that LV-Apelin (#4) was the most efficient shRNA, in which the RNA level of apelin was decreased by more than 70% (data was not shown). Then, we tested the knockdown efficiencies of LV-Apelin in pericytes. When MOI is 100, the result of immunofluorescence staining showed that interference efficacy achieved 90% (Figure 4(a)). After that, the qRT-PCR in pericyte revealed the apelin level was decreased by 75% in LV-apelin group compared to LV-GFP control group (*P* < 0.01) (Figure 4(b)). In a similar way, western blot demonstrated that the apelin level was downregulated by 64% (*P* < 0.05) (Figures 4(c) and 4(d)).

### 3.5. Apelin-Stimulated Cell Proliferation and Migration in Normoxia

**Figure 5:**
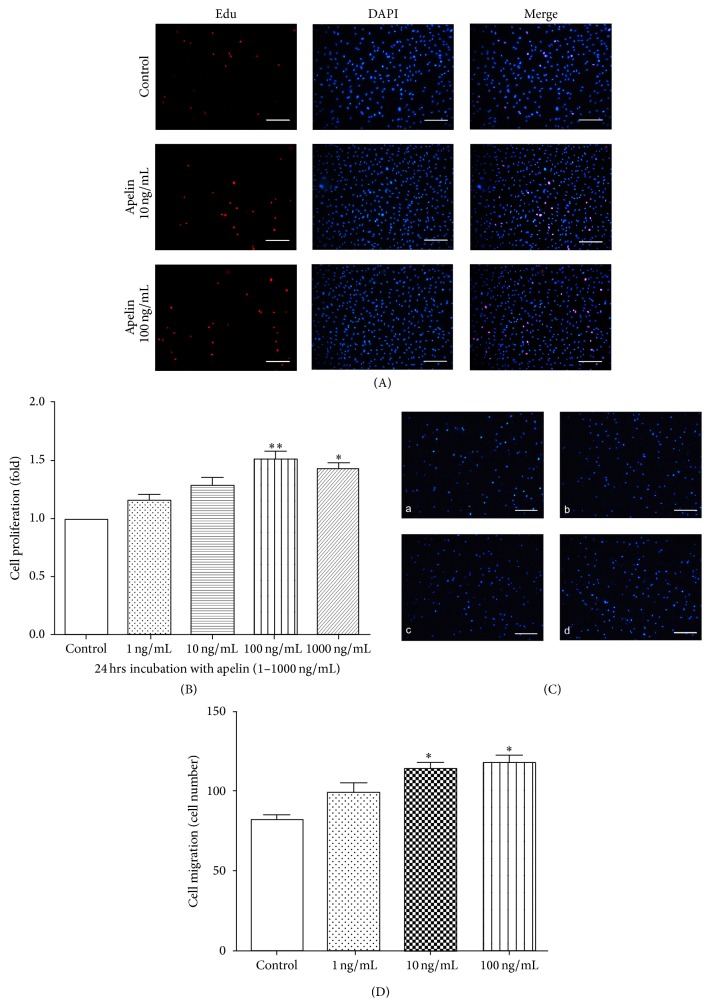
Effect of apelin on cell viability and migration under normoxia. (A) The Edu proliferation assay. Apollo staining (red) represents proliferating cells, and DAPI (blue) staining nuclei. Compared with the control group, the number of proliferating cells treated with apelin (10 or 100 ng/mL) increased significantly. Scale bar = 200 *µ*m. (B) The folds of apelin-treated cell viability compared with the control group (^*∗*^ *P* < 0.05, ^*∗∗*^ *P* < 0.01 versus untreated control); (C) and (D) pericyte migration in response to apelin treatment was measured using the transwell assay (a: control; b: 1 ng/mL; c: 10 ng/mL; d: 100 ng/mL, ^*∗*^ *P* < 0.05 versus untreated control). The data are expressed as means ± standard deviation (SD). Scale bar = 200 *µ*m.

Experiments were performed to evaluate whether apelin had any effect on pericytes proliferation and migration in normoxia. Pericytes were incubated with apelin at different concentrations (1, 10, 100, and 1000 ng/mL) for 24 h. Among the various concentrations, MTS assay results show that 100 ng/mL group significantly increased pericytes viability, compared with the control groups (Figure 5(B)) (100 ng/mL versus control, ^*∗∗*^ *P* < 0.01; 1000 ng/mL versus control, ^*∗*^ *P* < 0.05).

Edu experiment was used to detect pericyte proliferation at different concentrations of apelin (10 ng/mL and 100 ng/mL). The number of proliferative cells was significantly higher in the apelin-treated group, compared with the control group (Figure 5(A)).

In the cell migration assay, cells were measured in a modified Boyden Chamber in which pericytes migrated through a porous membrane. The mean number of migrated pericytes incubated with apelin (1–100 ng/mL) was significantly higher than the mean number of the control group (*P* < 0.05) (Figures 5(C) and 5(D)).

### 3.6. The Effects of Apelin and Lentivirus Knockdown Apelin for Cell Proliferation and Migration in Hypoxia

**Figure 6:**
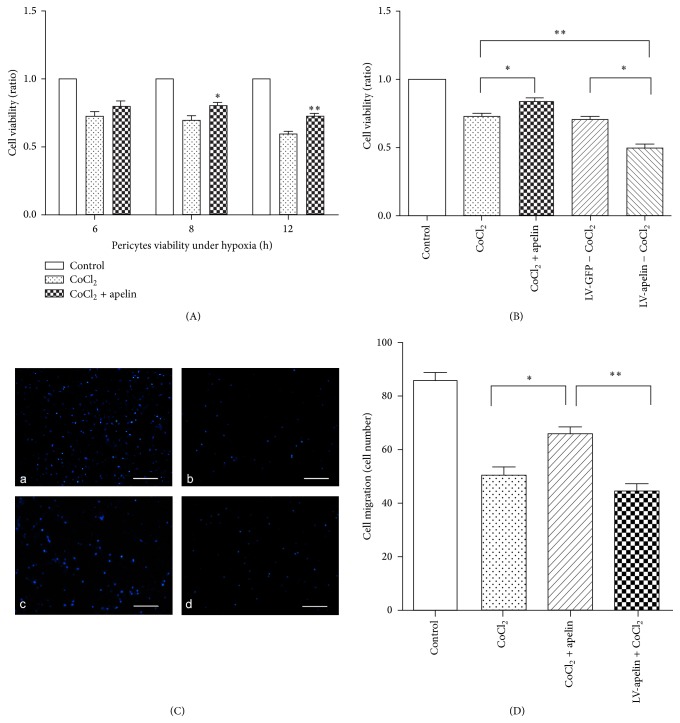
Effect of apelin on cell viability and migration under hypoxia. Cell viability was evaluated by MTS assay and migration was assessed with a transwell cell chamber. (A) In hypoxic pericytes, the viability of cells stimulated by apelin was significantly enhanced during 12 h (8 h versus CoCl_2_^*∗*^ *P* < 0.05; 12 h versus CoCl_2_^*∗∗*^ *P* < 0.01). (B) Viability of pericytes treated with apelin and LV-apelin knockout under hypoxia. Compared with the CoCl_2_ group, viability was significantly increased in the apelin group (*P *< 0.05). Moreover, cell viability was significantly reduced in the LV-apelin knockout group (LV-GFP versus LV-apelin,* P* < 0.05; CoCl_2_ versus LV-apelin,* P* < 0.05). (C) and (D) Pericyte migration induced by apelin under hypoxia (a: control; b: CoCl_2_ 150 *µ*mol; and c: CoCl_2_ 150 *µ*mol + apelin 100 ng/mL). The number of migrated cells per HPF is shown. Apelin versus CoCl_2_^*∗*^ *P* < 0.05. Scale bar = 200 *µ*m.

Furthermore, we carried out experiment to study the effect of apelin for pericyte under hypoxia. We found that the viability of pericytes incubated with CoCl_2_ was decreased obviously time dependently. The viability of pericytes was reduced by 27% at 6 h and by 40% at 12 h after stimulation by 150 *µ*mol/L CoCl_2_, respectively. However, the viability of pericytes stimulated by apelin was significantly enhanced, compared with the CoCl_2_ group (8 h versus control ^*∗*^ *P* < 0.05; 12 h versus control ^*∗∗*^ *P* < 0.01) (Figure 6(A)).

Our study showed that under hypoxia the viability of cells treated with apelin was significantly increased. Meanwhile, the viability of cells in the LV-apelin knockout group was significantly reduced (CoCl_2_ versus apelin, *P* < 0.05; LV-GFP versus LV-apelin, *P* < 0.05; CoCl_2_ versus LV-apelin, *P* < 0.01), which suggests that apelin can stimulate pericyte viability (Figure 6(B)). We also detected migration of pericytes under hypoxia. Compared with the control group, the mean number of migrated pericytes under hypoxia and combination with LV-apelin knockdown decreased significantly. However, in the group treated with apelin, the mean number of migrated pericytes was increased under hypoxia (Figures 6(C) and 6(D)).

### 3.7. Apelin Protected Pericytes against Apoptosis Induced by Hypoxia via Bcl-2/Bax Restoration and Caspase 3 Pathway

**Figure 7:**
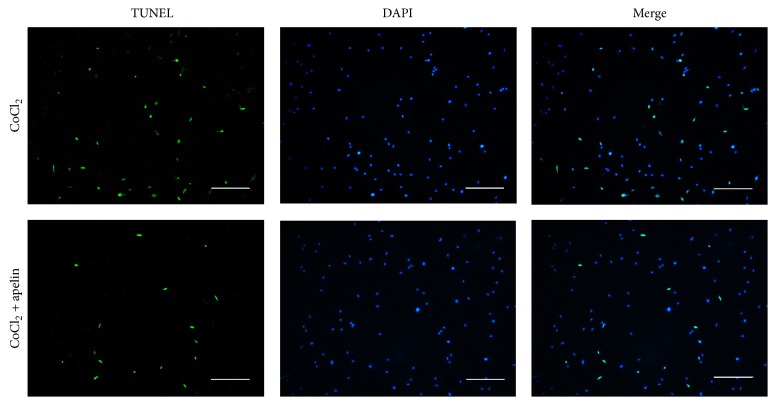
TUNEL staining was used to evaluate hypoxia-induced cell death. Cells were exposed to 100 ng/mL apelin for 12 h and then exposed to hypoxia for 12 h. Apoptotic nuclei were visualized by TdT-mediated dUTP nick end-labeling (TUNEL). Scale bar = 200 *µ*m.

In the cell viability experiment, hypoxia resulted in a 27% decrease in pericyte viability. However, cell viability increased significantly in pericytes pretreated with 100 ng/mL of apelin for 12 h. To further evaluate the effects of apelin on cell death, we used TUNEL staining to detect DNA fragmentation and cell death in hypoxia-treated pericytes with and without apelin treatment. We pretreated pericytes with apelin for 12 h and then exposed these pericytes to hypoxia for 12 h. In the percentage of apoptotic cells showing green, approximately 30% of cell death was blocked by the apelin treatment (Figure 7).

**Figure 8:**
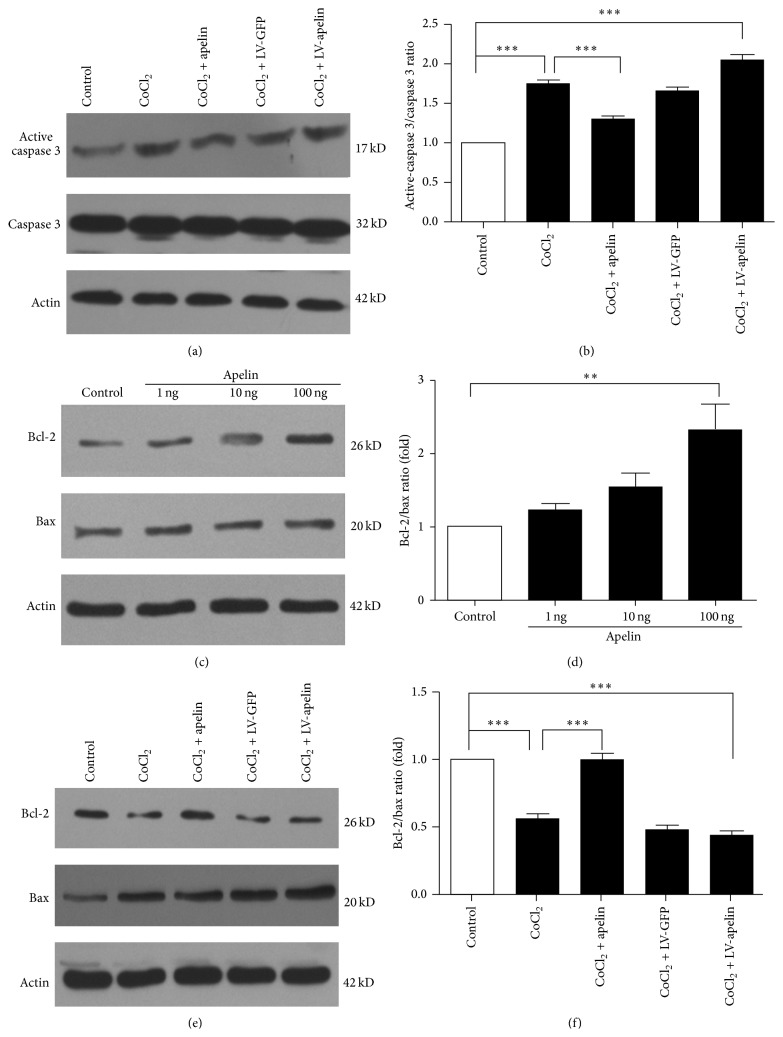
Effect of apelin on hypoxia-induced apoptosis in rat retinal pericytes. (a) Active-caspase 3 protein level significantly increased 12 h after hypoxia injury (*P* < 0.001). Apelin significantly decreased its levels after hypoxia injury (*P* < 0.01). (c and e) Effects of apelin on Bcl-2 and Bax protein expression in rat retinal pericytes. Cells were incubated with apelin and LV-apelin knockout under hypoxia. Western blot analysis was quantitated by densitometry of autoradiographs, and the relative mean ratio of Bcl-2/Bax was increased in apelin group (*P* < 0.001 versus con.) and reduced in Lv-apelin knockdown group (*P* < 0.001 versus con.).

Active-caspase 3 protein is one of the key executioners of apoptosis. As shown in Figure 8, active-caspase 3 protein level significantly increased 12 h after hypoxia injury (*P* < 0.001, compared with the control group). Administration of apelin significantly decreased its levels after hypoxia injury (*P* < 0.01, compared with hypoxia group). The expression of active-caspase 3 in hypoxia combination with LV-Apelin knockdown was similar to hypoxia group and its levels significantly increased (*P* < 0.01, compared with control group) (Figures 8(a) and 8(b)).

Likewise, we also detect bcl-2 and Bax expression in pericyte. Western blot analysis showed that apelin dose-dependently induced Bcl-2 protein expression and downregulated Bax protein expression in pericytes (Figures 8(c) and 8(d)). The antiapoptotic effect of apelin was through increased expression of Bcl-2 and reduced expression of Bax. In hypoxia group, the ratio of Bcl-2/Bax decreased 36% and LV-apelin group has a similar ratio (*P* < 0.001, compared with con. group). However, the ratio of Bcl-2/Bax in treatment with apelin group was significantly increased (*P* < 0.001, compared with hypoxia group) (Figures 8(e) and 8(f)).

## 4. Discussion

Apelin interacts with its specific receptor APJ, has multiple biological activities, and had been characterized in various tissues [31]. Previously, we proved that vitreous concentrations of apelin were significantly higher in the proliferative diabetic retinopathy (PDR) group. Likewise, apelin and APJ also colocalized with endothelial cells maker CD 31 in PDR [30]. In the present study, we further demonstrated that APJ was strong expressed in fibrovascular membranes of the PDR and was colocalized with pericytes. Therefore, apelin/APJ system was possibly involved in the pathological progression of PDR. However, the effect of apelin on apoptosis of primary retinal pericytes remains unknown. In order to study the effects of apelin in pericyte, primary rat pericytes were used here. Based on the current results, we proved the expression of apelin and APJ in pericytes and demonstrated that apelin and APJ are upregulated in hypoxia cultured condition. Knockdown of apelin inhibits proliferation and migration of pericytes. Moreover, exogenous recombinant apelin effectively prevented hypoxia-induced apoptosis through downregulating the expression of active-caspase 3 and increased the ratio of Bcl-2/Bax in pericyte. These results establish the foundation for further study of diseases associated with ischemia and hypoxia.

As we all know, rodent is similar to human in genetic background. In previous published studies, primary cultured pericytes were mostly originated from bovine retina, which restricted further* in vivo* studies [32]. In the present study, we established a rodent- (rat-) based primary pericyte* in vitro* model. By using a magnetic beads isolation method, we obtained primary rat retinal pericytes successfully in the purity of 90%. As for marker of pericytes, alpha smooth muscle actin (*α*-SMA), tropomyosin desmin, nestin, sulfatide or nerve/glial antigen-2 (NG2) proteoglycan, platelet-derived growth factor receptor-B (PDGFR-B), aminopeptidase N (CD13), and the regulator of G-signaling 5 (RG5) are common pericyte markers [4, 33]. However, no single entirely pericyte-specific marker is known to date, and all markers currently used are dynamic in their expression and may be up- or downregulated in conjunction with developmental states, pathological reactions, and* in vitro *culturing conditions [4]. For example, pericytes on normal capillaries typically express desmin, but not SMA, whereas smooth muscle cells on arterioles and pericytes on venues are immunoreactive for both [34]. Therefore, we select three markers that sufficiently identify pericytes in order to obtain highly pure rat retinal pericytes. In present study, pericytes uniformly expressed the cellular markers PDGFR-*β*, NG2 and desmin. Our results are consistent with Liu's study, who also proved these markers expressed in pericytes isolated from rats by mechanical morcellation and collagenase digestion [29].

Apelin/APJ is localized in a wide variety of tissues, including the endothelial cells of the primary blood vessels, neurons, and oligodendrocytes [18]. The lines of evidence show that apelin exerts its biological functions through its interaction with APJ. Knockout of apelin or APJ leads to the inhibition of both hypoxia-induced endothelial cell proliferation* in vitro* and hypoxia-induced vessel regeneration in the caudal fin regeneration of Fli-1 transgenic zebrafish [35]. Therefore, location of APJ in cells or tissues is very important with regard to apelin exerting its diverse functions. In the present study, through immunofluorescence staining, we first confirmed the expression of APJ in pericytes and hypoxia-induced upregulation of apelin and APJ. The results of this study showed that the expression of APJ was positive in pericytes, which is essential for apelin/APJ system and plays a role in pathological and physiological condition. This suggested that apelin might be involved in pericyte physiology and pathology.

Apelin was shown to have angiogenic activity in retinal endothelial cells, both* in vitro* and* in vivo* [18]. In our previous studies, we showed that apelin can enhance proliferation and migration of Müller cells and RPE cells [19, 21]. Eyries identified apelin as a hypoxia-inducible factor-1 (HIF-1) target gene and demonstrated that, under hypoxia, HIF-1 binds to the first intron of apelin, leading to upregulation of apelin expression [35]. In the present study, we observed that the viability and migration of pericytes incubated with various concentrations of apelin were enhanced. Under hypoxia exposure, pericytes viability decreased significantly with time-dependent manner and apelin can protect pericytes viability. Furthermore, knockdown of apelin led to a significant decrease in pericyte viability. These results further support the hypothesis that apelin is sensitive to hypoxia, playing a key role in hypoxia-induced pericyte proliferation and migration.

Many* in vitro* and* in vivo* insults, such as hypoxia and ischemia, trigger mixed cell death composed of both necrosis and apoptosis [31, 36]. Hypoxia-induced Bax upregulation, Bcl-2 downregulation, and caspase 3 activation in variety of cells were reversed by HIF-1 overexpression and lead to the acquisition of antiapoptotic properties [37–39]. The ratio of antiapoptotic to proapoptotic proteins, especially the Bcl-2/Bax ratio, determines susceptibility to apoptosis [40]. We therefore investigated whether these pathways were involved in the antiapoptotic effects of apelin in pericytes. Our result was consistent with previous studies, which indicated that Bcl-2/Bax apoptotic signaling pathways mediate the protective effects of the apelin/APJ system in vascular smooth muscle cells and osteoblasts [17, 24]. Caspases, cysteine proteases with aspartate specificity, are important mediators of apoptosis. Caspase 3 is effector caspase that is responsible for cleaving nucleases in addition to cellular substrates. We also revealed that apelin reduced caspase 3 activity, which suggests that apelin inhibits pericyte apoptosis through regulation of activity of caspase 3 and Bcl-2/Bax expression. Therefore, there is a growing consensus that apelin may be a promising therapeutic target against hypoxia/ischemia in the future.

In conclusion, this study demonstrated that apelin/APJ was expressed in PDR patient's membranes and in rat retinal pericytes. Apelin can protect pericytes against hypoxia-induced apoptosis through regulation of activation of caspase 3 and Bcl-2/Bax expression. These results indicated that apelin could be a potential therapeutic target for retinal angiogenic diseases.

## 5. Conclusion

Pericytes are a population of cells that are involved in normal vessel architecture and contraction and regulated blood flow. Hypoxia causes decreasing of pericytes viability in a time-dependent manner and induced pericytes apoptosis. However, apelin regulated function of pericytes under hypoxia inversely in a concentration-dependent manner and effectively prevented hypoxia-induced apoptosis through downregulating active-caspase 3 expression and increasing the ratio of Bcl-2/Bax.
